# Advances in immunological sorting of X and Y chromosome-bearing sperm: from proteome to sex-specific proteins

**DOI:** 10.3389/fvets.2025.1523491

**Published:** 2025-03-12

**Authors:** Linfeng Bai, Yue Zhao, Yang Zhou, Yongli Song, Hao Xiao, Gaoping Zhao, Zhigang Wang, Xihe Li

**Affiliations:** ^1^State Key Laboratory of Reproductive Regulation and Breeding of Grassland Livestock, School of Life Sciences, Inner Mongolia University, Hohhot, China; ^2^Research Center for Animal Genetic Resources of Mongolia Plateau, Inner Mongolia University, Hohhot, China; ^3^National Center of Technology Innovation for Dairy Industry, Hohhot, China; ^4^College of Basic Medicine, Inner Mongolia Medicine University, Hohhot, China; ^5^Inner Mongolia SaiKexing Institute of Breeding and Reproductive Biotechnology in Domestic Animal, Hohhot, China

**Keywords:** sperm sorting, immunological approach, sex-specific protein, sexing technology, spermatozoa proteome

## Abstract

Sex determination is the developmental assignment that results from genetic factors. The sexual characters were the specific manifestations of male and female individuals under stimulation of sexual hormonal production. The fusion of an oocyte with an X chromosome-bearing sperm will lead to a female (XX), while fusion with a Y chromosome-bearing sperm will develop into a male (XY) in mammals. Sexing technology has been developed to fertilize eggs with sorted sperm, producing offspring of the desired sex. Sperm sorting enables the sex pre-determination of offspring via *in vitro* fertilization (IVF) or artificial insemination (AI) in domestic animals. Flow cytometric sorting of X and Y sperm is widely considered the most applied method for sperm sorting and has been commercially applied in cattle. However, a non-invasive, immunological method for screening X and Y sperm is considered to be a feasible approach. This review summarizes the current knowledge and techniques of sperm immunological sorting, including the preparation of antibodies, application of immunomodulators, and immunoisolation. Additionally, we focus on identifying sex-specifically expressed proteins in X and Y sperm through proteomic analysis, and verifying the sex-specific proteins using experimental techniques. Furthermore, several housekeeping proteins as loading control were discussed in immunoblotting of sperm proteins. Immunological sorting of X and Y sperm could provide a convenient, cost-effective, and highly efficient technique that can improve economic benefits and achieve an advanced level of sexing technology. This review provides insight into immunological sorting of sperm and the pre-determination of sex in farm animals.

## 1 Introduction

Spermatozoa sex-sorting technology has been extensively studied over the past decades, and several technological models have been developed for separating X- and Y-chromosome-bearing sperm in mammals. Previous studies have proposed several approaches to separate X and Y chromosomal sperm based on the substantial differences between X and Y spermatozoa, including DNA content, shape and size, surface chemistry, motility, zeta potentials, pH, and unique proteins and immunological markers (1–3). Currently, the most effective and practical method for quantitatively distinguishing and separating X and Y sperm is flow cytometry-based sex-sorting, which relies on the different DNA content of the X- and Y-chromosomal sperm (2, 4). This technique is applicable for sperm separation for several animals, such as rabbit, cattle, goat and sheep (4, 5). However, only the separation of cattle sperm has reached the stage of commercial application in family farms and ranches. Due to the current limitations of sperm sorting: low sex-sorted sperm efficiency, sperm damage, and high costs with flow cytometry sorting, it’s crucial to improve sperm sorting approaches to enhance offspring reproduction efficiency and high-level sex pre-determination in domestic animals. The immunological approach for X and Y sperm sorting offers a feasible, economical and effective technique with potential commercialization, particularly beneficial for small family farms and ranches.

Immunoisolation of cells refers to a cell separation technique based on the ability of cell surface antigens to bind to specific antibodies. In a broader sense, it also includes the separation of cells treated with immunomodulators. Immunological separation has been widely applied in animal somatic cell research and in diagnosing and treating human diseases (6, 7). This includes agglutination reactions, immunoadsorption, and complement-mediated cytotoxicity or complement fixation reactions. Immunoagglutination is the process in which particulate antigen or sensitized carrier particles covered with soluble antigen bind to corresponding antibodies, resulting in visible agglutination. Such reactions can be categorized as direct (particulate antigen) or indirect (soluble antigen) immunoagglutination. Agglutination reactions occur when cells with specific antigens bind to corresponding antibodies, while other cells remain unaffected. These free-floating cells can be separated by external force or through their inherent motility (8, 9). Immunoadsorption involves immobilizing an antibody onto a particular carrier, such as magnetic beads. Cells with a specific surface antigen become attached via specific antigen–antibody binding when cells interact with this antibody-carrier complex, while cells without this specific surface antigen remain unbound. The attached and unattached cells can be separated by subjecting this system to external forces, such as magnetic fields or flowing fluids (6, 10). Complement-mediated cytotoxicity or complement fixation reaction refers to using antibodies to bind to cells with specific surface antigens, resulting in the formation of an antigen–antibody complex. Subsequently, a complement system (e.g., animal serum) is introduced to react with the antigen–antibody complex. Upon complement activation, it attacks the cells with antigens, inducing cell damage and loss of function in these cells. The antibody involved in the complement fixation reaction is primarily IgG, which contains a complement binding site (the Fab) in the Fc segment (CH2 region) of its heavy chains (11–13). Of the three immunological approaches mentioned above, the most critical challenge is to obtain antibodies against cell surface specific antigens.

**Figure 1:**
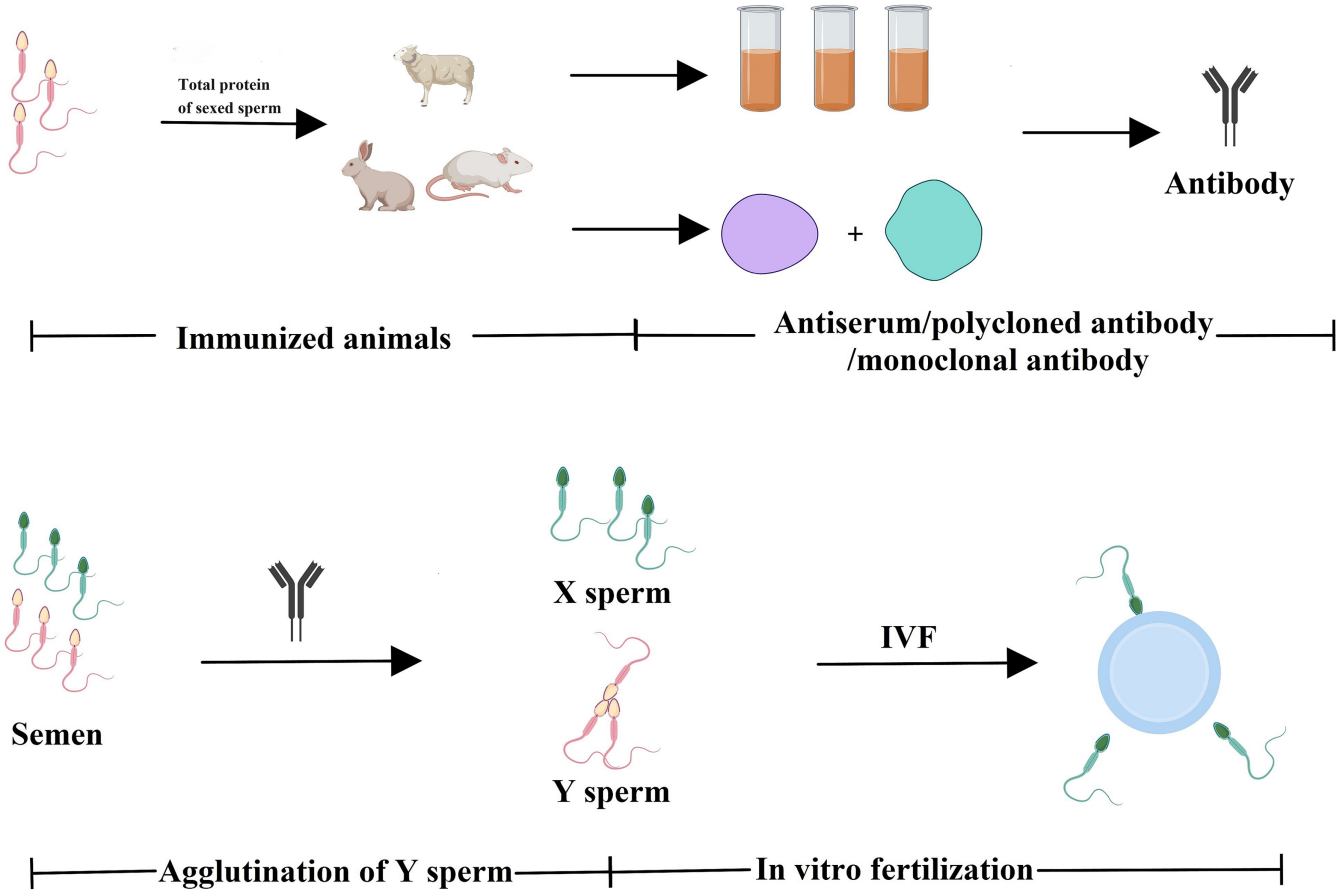
Total proteins of sexed sperm were used to immunize animals to generate antibodies against X- or Y-chromosome-bearing sperm. Animals (e.g., mice, rabbits, and sheep) were immunized with total sperm proteins to generate antisera, which can be purified to obtain polyclonal antibodies. Alternatively, spleen cells that produce antibodies can be isolated from immunized animals, and then monoclonal antibodies are then obtained through hybridoma technology. The antibodies can be used to separate X and Y sperm via immunological approaches (e.g., agglutination reactions). Created with MedPeer (medpeer.cn).

**Figure 2:**
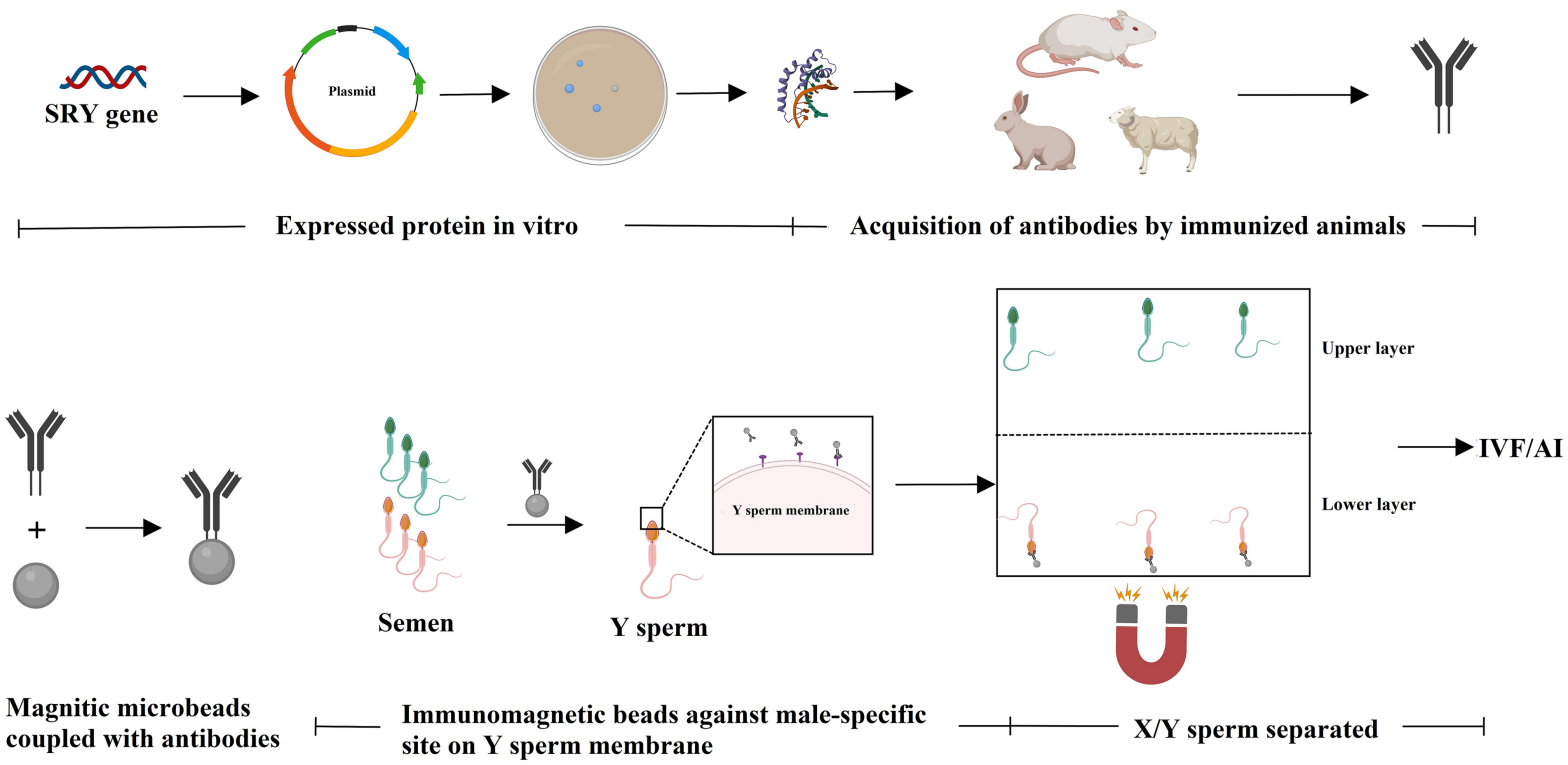
Known sex-specifically expressed protein was used to immunize animals to generate antibodies against X- or Y-chromosome-bearing sperm. The unique protein that expressed in Y sperm (e.g., SRY) is used to immunize animals to generate antibodies against Y-chromosome-bearing sperm. The antibodies can be used for sperm separation via immunological approaches (e.g., immunoadsorption). Created with MedPeer (medpeer.cn).

**Figure 3:**
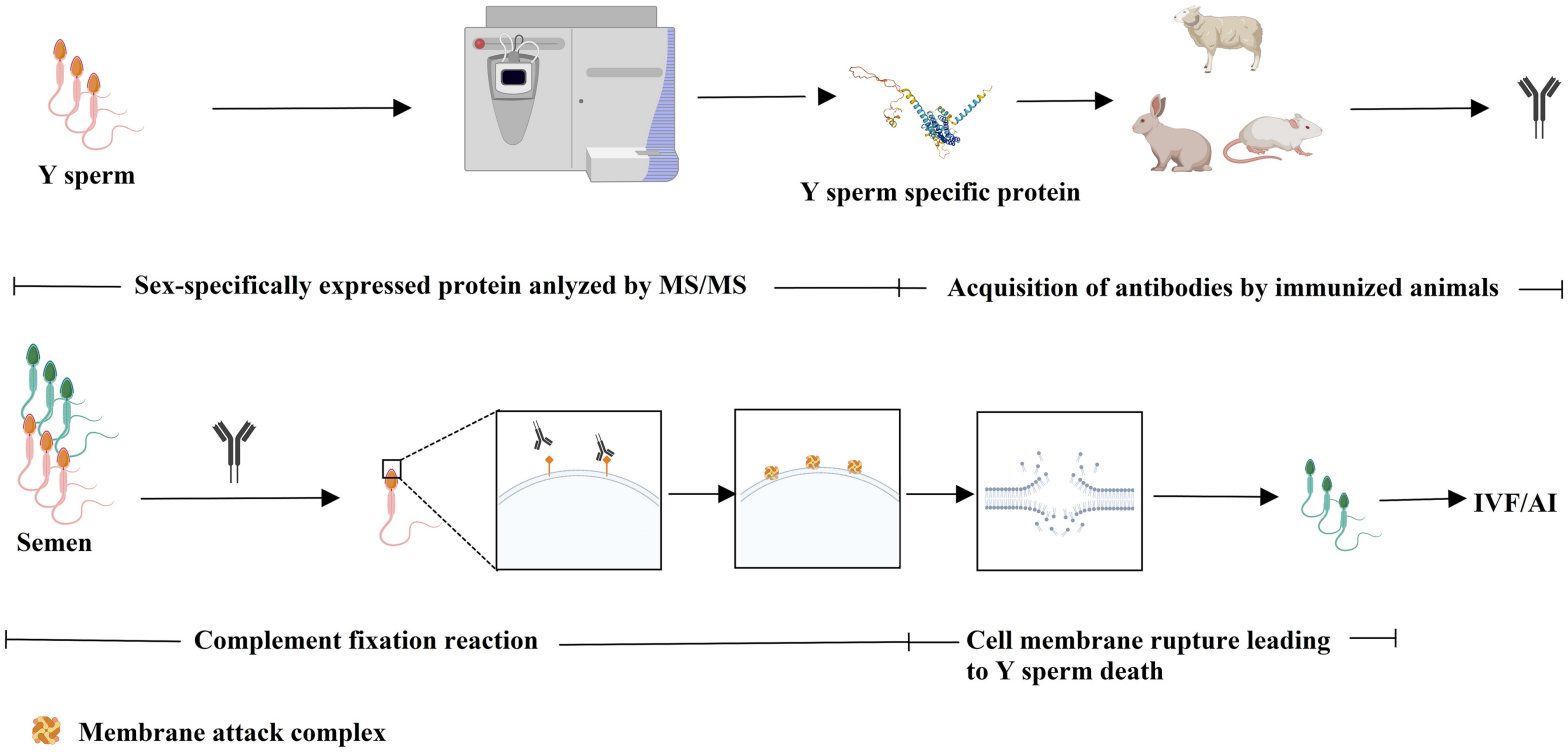
Comparative proteomic analysis was used to identify sex-specifically expressed protein to generate antibodies via immunizing animals for sperm sorting. Unique proteins were identified from proteome of X or Y sperm. The sex-specifically expressed protein can be utilized to generate antibody for sperm sorting via immunological approaches (e.g., complement fixation reaction). Created with MedPeer (medpeer.cn).

**Figure 4:**
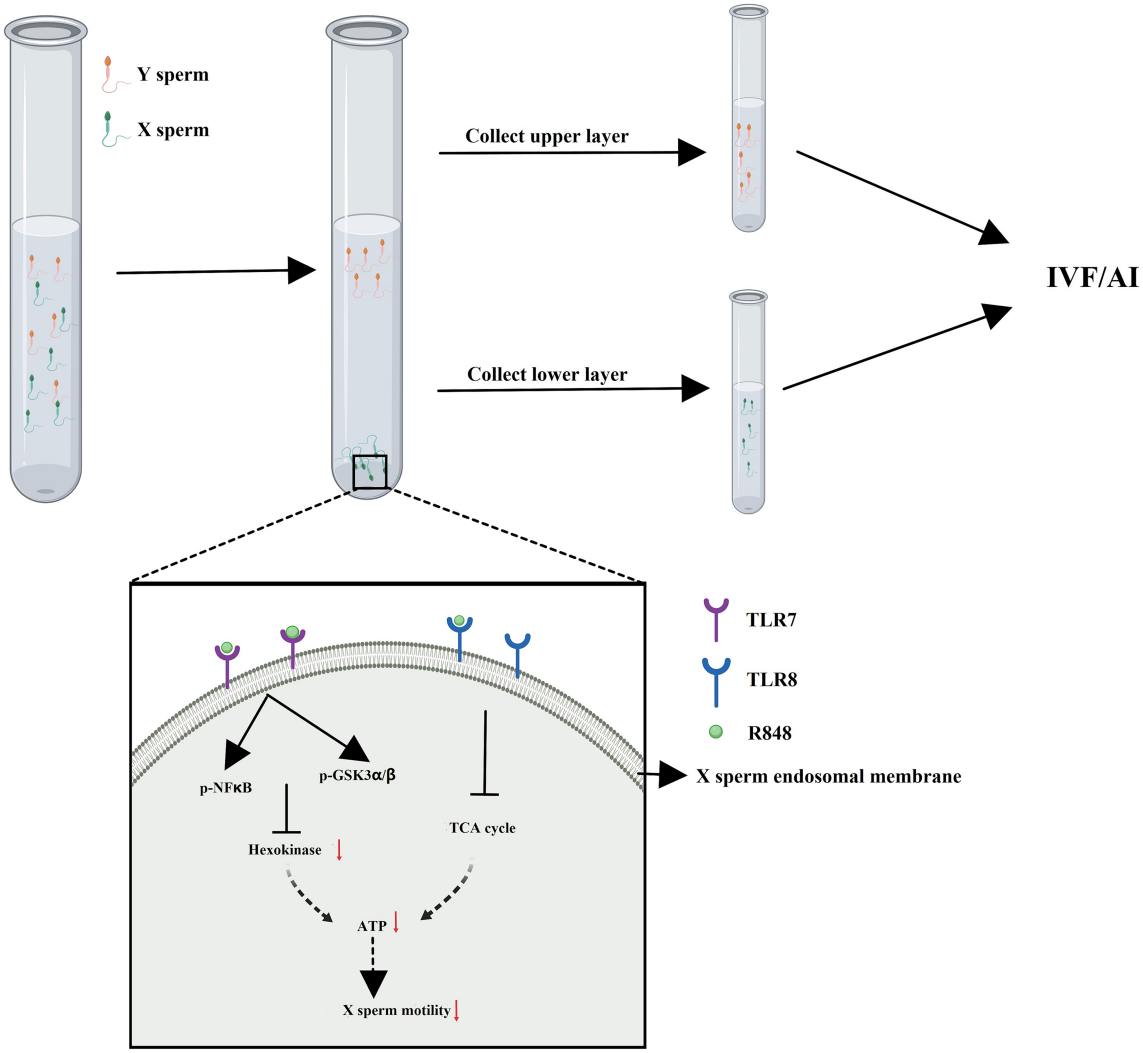
Immunomodulator treatment (R848) combined with sperm swim-up method to separate X and Y sperm. R848 is a specific dual agonist for TLR7/8 specifically expressed in X sperm. The motility of X sperm saw a decline and presented in the lower layer of sperm swim-up buffer due to R848 treatment. Created with MedPeer (medpeer.cn).

The immunological approach for sorting X and Y sperm is also a kind of cell separation technology that relies on the principle of specific antigen–antibody reactions. There are three main technical roadmaps that can be used to generate antibodies and subsequently perform immunological sorting of X and Y chromosome-bearing sperm: (i) Animals (e.g., mice, rabbits, and sheep) were immunized with total sperm proteins to generate antiserum, which can then be purified to obtain polyclonal antibodies. Alternatively, spleen cells that produce antibodies can be isolated from immunized animals, and monoclonal antibodies against X or Y sperm are then obtained through hybridoma technology. Additionally, single-chain antibody variable fragments (scFv) can be synthesized to sort X and Y sperm (14–18) (Figure 1). (ii) Another approach takes advantage of unique proteins that are expressed in X or Y sperm, such as the sex-determining region Y (SRY) and H-Y, which was once considered a Y-specific protein (19–23). Antibodies against the SRY antigen were used for sperm separation (Figure 2). (iii) Proteomic analysis was used to identify sex-specifically expressed membrane proteins on X and Y sperm. These sex-specifically expressed proteins can be utilized to generate antibodies for sorting X and Y sperm (24–26) (Figure 3). Of these three methods, the first two have demonstrated positive separation results (14–18, 22, 23), while the third method has reported discoveries of unique proteins for X and Y sperm, along with their verification (26). In addition, achievement has been made in sorting X and Y sperm using immunomodulator treatment (R848) combined with the sperm swim-up method (Figure 4), which based on the specific expression of toll like receptor 7/8 (TLR7/8) in X sperm (27, 28).

Among the available scientific and patent literature, studies conducted by scholars at Chiang Mai University in Thailand (14–16, 29) and Nuri Science Company in South Korea (17, 18, 30, 31) have achieved obvious results in the immunoseparation of cattle sperm. Recently, Soleymani and colleagues from the Kermanshah University of Medical Sciences in Iran reported on antibodies that can specifically bind to bovine Y-chromosome bearing sperm (22, 23). At present, no membrane proteins have been identified for sperm sorting, and the reported examples of immunological sorting are not as commercialized as flow cytometry. Sperm membrane proteomic analysis may serve as a candidate for finding unique proteins for immunological sorting of X- and Y-chromosome bearing sperm in livestock.

## 2 Antibodies and single-chain antibody variable fragments for sperm sorting

In 1954, Wilson and Ruimke, respectively, reported the discovery of sperm agglutinins or autoantibodies against spermatozoa in sterile men (32, 33). Subsequently, Isojima also identified sperm-immobilizing antibodies in the sera of women with unexplained sterility (34, 35). The interaction of sperm with female serum or cervical mucus *in vitro* can result in head-to-head (36, 37), tail-to-tail, and head-to-tail agglutination (37–39). Upon binding with complement, anti-sperm antibodies exhibit the characteristics of cytotoxic antibodies and have cytotoxic effects on sperm (40). In 1975, Kohler and Milstein reported the creation of cell lines which are made by fusion of a mouse myeloma and mouse spleen cells to produce monoclonal antibodies (41). Significant advancements have been made in generating monoclonal antibodies using hybridoma technology since then. These advancements have found applications in clinical disease management and various immunological detection techniques. The development of anti-sperm monoclonal antibodies through hybridoma technology started in the early 1980s (42–45). These monoclonal antibodies were subsequently employed to detect and validate the expression of surface antigens on human, mouse, and cattle sperm (46–48), investigate the influence of the antibodies on capacitation, acrosome reaction, and antifertility effects (49–51). By the late 1990s, efforts were underway to explore the potential of immunological sorting of sperm, specifically the production of antisera used for sorting bovine sperm (52).

A group from Chiang Mai University in Thailand has reported a series of work on an immunological approach for sorting bovine X and Y sperm since 2010. Initially, the focus was on producing antibodies against Y sperm. Subsequently, the male-specific antibody was used in a cytotoxic reaction to selectively increase X sperm in whole sperm by lysing Y sperm. Furthermore, single-chain antibody variable fragments (scFv) were synthesized against Y-chromosome-bearing sperm, and Y sperm were separated using the scFv antibodies coupled with magnetic microbeads (14–16, 53–55). Pattanawong et al. (53) immunized mice with dairy bull spermatozoa (conventional semen), and then isolated splenocytes which produced antibodies to the Y-bearing spermatozoa. The splenocytes and mouse myeloma cells were fused, and a hybridoma cell 1F9 was cloned secreting mAb-1F9, a specific Y-bearing sperm monoclonal antibody (53). In 2014, Dumrongsri et al. produced monoclonal antibodies P1C2B8 and P1C2C9 using the same approach. These antibodies presented greater binding affinity to male bovine white blood cells compared to female white blood cells (54). Subsequently, Dumrongsri et al. conducted complement-mediated cytotoxicity tests identifying those monoclonal antibodies (P1C2B8 and P1C2C9) could effectively reduce the number of viable Y sperm, thereby modifying the X/Y sperm ratio (55). Furthermore, using MAb-1F9 combined with the cytotoxicity reaction (IC-sexed), the percentage of live Y-chromosome bearing sperm in the IC-sexed semen was lower than that of X-chromosome-bearing sperm. As a result, 74.29% of the offspring born after artificial insemination (AI) with IC-sexed semen were female calves (14). In 2022, Thaworn et al. from the same group used the overlap PCR to clone gene fragments from the variable regions of heavy and light chains of antibodies using the hybridoma cell line 1F9 established by Pattanawong et al. Subsequently, a soluble scFv was produced by connecting these fragments through a linker and expressing them in prokaryotic cells. The scFv antibody presented a high affinity for Y-bearing sperm and low cross-reactivity with X-bearing sperm (15), making it suitable for X/Y sperm separation. In the same year, this scFv antibody was coupled with magnetic microbeads to develop an immunomagnetic beads and successfully enhancing the separation efficacy and achieving X/Y sperm enrichment rates of 82.65 and 81.43%, respectively (16). The team from Chiang Mai University pursued a clear technical approach: initially producing hybridoma monoclonal antibodies against Y sperm and subsequently introducing the single-chain antibody scFv, and sorting sperm with these antibodies coupled with magnetic microbeads. The application of these approaches was demonstrated in bovine X/Y sperm sorting and the pre-determination of sex in bovines.

Another example of immunological sorting is the bovine sperm sorting kit produced by Nuri Science, a company based in South Korea. The WholeMom (female cow product) is utilized for sorting bovine X/Y sperm, with a 90% success rate in selecting the desired sex (F/M) (18). Chowdhury et al. from the National Institute of Animal Science in South Korea used the WholeMom kit to sort bovine sperm, followed by the generation of embryos through *in vitro* fertilization (IVF). The bovine embryos exhibited high-cleavage embryo rate and blastocyst formation rate in X-sperm sorted groups, with female embryos accounting for 81.0% of those produced *in vitro*. The proportion of female offspring reached 76% for artificial insemination using WholeMom-treated sperm (30). Further research by Sidrat and colleagues from Gyeongsang National University in Korea found that sperm separated using this Y sperm-specific antibody exhibited variations in early embryonic developmental kinetics and epigenetic characteristics between male and female bovine embryos *in vitro* (31). Patent records indicate that Nuri Science produced scFv antibody for Y-chromosome bearing sperm, and subsequently sought international patent protection for the antibody and its related applications (17). However, the specific antibody used in the WholeMom sex sorting kit and details regarding Y sperm surface antigens remain undisclosed (18). Nevertheless, WholeMom, a female cow product produced by Nuri Science, is the first commercial product in the field of immunological sorting of bovine X and Y-chromosome bearing sperm, with promising future potential.

## 3 Antibodies of anti-H-Y antigen and anti-SRY antigen for Y-chromosome bearing sperm

The technical approach for separating sperm based on Y sperm specific proteins involves two proteins: the male specific minor histocompatibility Y antigen (H-Y antigen) and the sex-determining region on the Y chromosome (SRY) protein, with the coding genes for both located on the Y chromosome in humans and mice. Y-chromosomal antigenicity was first described for skin grafts in the 1950s (56, 57). In 1971, Goldberg et al. detected H-Y antibodies in mouse serum (58). Subsequently, Bennett et al. used anti-H-Y serum-treated semen for artificial insemination and observed an increase in the proportion of female offspring (19). The H-Y antigen has been found on the surface of sperm from various animals and is perceived as a male-specific antigen associated with testicular differentiation and sex determination. It was once thought to provide a potential means to differentiate between X and Y sperm (20, 59, 60). However, several groups presented different findings. Specifically, McLaren et al. revealed that mice could develop a male phenotype even in the absence of the H-Y antigen. This result suggests that the antigen is unlikely to be responsible for testis determination (61). Ali et al. conducted bovine sperm sorting using anti-H-Y monoclonal antibodies, and the results showed equal proportions of X and Y sperm (62). Several researchers have found that the H-Y antigen is present in X spermatozoa and does not separate X/Y spermatozoa (63, 64). Consequently, interest in the H-Y antigen as the primary focus of sperm sorting diminished, and no novel products or successful applications emerged.

In 1990, the Lovell-Badge group reported a series of work that introduced the discovery of the SRY gene in humans and mice. This gene is located on the Y chromosome and plays a critical role in testicular differentiation and sex determination (65–68). Subsequent work demonstrated that the SRY gene possesses a DNA-binding domain and exhibits characteristics of an activating transcription factor (69–72). It is believed to be located within the nucleus (73, 74). Over the past 30 years, most studies have primarily focused on its role in sex determination, with less attention given to its potential application in sperm sorting. Exceptionally, a 2011 report by Li et al. from Jilin University in China, revealed that the SRY protein is expressed at the acrosome of the bovine Y sperm head through immunofluorescence (75). Subsequently, Hashimoto et al. (21) from the Central Institute for Experimental Animals in Japan, achieved a male offspring proportion of 86.1% by application of mouse spermatozoa labeled with Cy3-SRY antibody conjugated for intracytoplasmic sperm injection (ICSI). Y sperm bind to SRY antibodies conjugated to magnetic beads (Mag) and are adsorbed to the bottom of the culture medium in the presence of a magnetic field. The supernatant of the medium was then used for *in vitro* fertilization (IVF) and embryo transfer (ET), resulting in a 67.3% proportion of female progeny (21). In 2019 and 2021, Mostafaie and Soleymani from Kermanshah University of Medical Sciences in Iran, reported that they immunized goats and mice with recombinant bovine SRY antigen. This resulted in the production of serum polyclonal antibody pAb and monoclonal antibody mAbSRY2, which are capable of specifically binding to Y sperm but not to X sperm (22, 23). These findings from Li et al. (75), Hashimoto et al. (21), and Mostafaie and Soleymani (22, 23) suggest that the SRY protein or a fragment of SRY, may be located on the cell surface of bovine Y sperm. This new subcellular localization of the SRY protein represents a surprising breakthrough and an extension of its function. These novel findings, particularly the cell membrane localization of SRY and the approach of using anti-SRY antibodies for sperm sorting, necessitate further experimental validation.

In summary, animals were immunized with either total protein from sexed sperm or with a Y-specifically expressed protein to produce antibodies. These antibodies were then used for immunological sorting of X and Y sperm. However, both of these approaches have limitations in their applications. Although the first approach is straightforward, the production of antisera is restricted by low antibody specificity. There are still uncertainties regarding monoclonal antibodies obtained by constructing hybridoma cell lines, such as high secretory specificity and high activity. The second approach relies on Y-specifically expressed proteins, and only SRY protein currently available. However, the question of whether SRY is localized on the surface of sperm in livestock and its potential application in sperm sorting via immunological approaches require further validation.

In any case, there is another important issue to be addressed with both methods - antibody specificity. Our review of the extant literature indicates a significant disparity in the efficiency of antibody-facilitated separation of X and Y spermatozoa. Notably, the monoclonal antibodies Mab-1F9 and WholeMom Product achieve separation efficiencies of 74.29 and 72.5%, respectively (14, 30). In stark contrast, the polyclonal antibody CY3-SRY demonstrates a markedly higher separation efficiency of 86.1% (21). These results reveal the instability of antibody-mediated isolation techniques for X and Y spermatozoa, thus emphasizing the need to optimize the antibodies used in such procedures. The instability associated with antibody-mediated separation of X/Y spermatozoa is likely to have a consequential impact on the fidelity of sex determination in resultant offspring. A summary of the application of various antibodies for immunological sorting of sperm is presented in Table 1.

**Table 1:** The antibodies and their efficiency for sperm sorting.

Species (spermatozoa)	Name	Efficiency of separation	Reference
Bovine	Mab-1F9	X, 74.29%	Thongkham et al. (14)
Bovine	Mab-1F9 scFv	X, 82.65%	Sringarm et al. (16)
Bovine	Mab-1F9 scFv	Y, 81.43%	Sringarm et al. (16)
Mouse	Anti-HY serum	Unknown	Bnnett et al. (19)
Mouse	Anti-HY serum and complement	X, 82%	Shelton and Goldberg (20)
Mouse	Cy3-SRY	Y, 86.1%	Hashimoto et al. (21)
Mouse	Mag-SRY	X, 67.3%	Hashimoto et al. (21)
Bovine	SRY	Unknown	Soleymani et al. (23)
Bovine	WholeMom Product	X, 81%	Chowdhury et al. (30)
Bovine	WholeMom Product	Y, 72.5%	Chowdhury et al. (30)
Bovine	PIC2B8	Unknown	Dumrongsri et al. (54)
Bovine	PIC2C9	Unknown	Dumrongsri et al. (54)
Bovine	H-Y	No significance	Ali et al. (62)

Mab, monoclonal antibody; scFv, single-chain fragment variable.

## 4 Finding unique proteins in X-, Y-chromosome bearing sperm via proteomic analysis

During testicular development and spermatogenesis, it has been proposed that adjacent haploid spermatids remain interconnected with neighboring cells through cytoplasmic bridges. These cytoplasmic bridges permit sharing of gene products between postmeiotic haploid spermatids. This mechanism can effectively decrease phenotypic differences between individual haploid sperm (76, 77). However, there are exceptions to the rule of complete sharing of transcripts between spermatids. In March 2021, Bhutani et al. reported that a significant number of mammalian genes are not completely shared across these bridges (78). The gene products that are not completely shared through spermatid cytoplasmic bridges may lead to differences in protein profiles between X and Y sperm, particularly in membrane protein profile (26). These differences result in the stable inheritance of sex-specific constitutive expression at the sperm level and serve as the theoretical foundation for the immunological sorting of sperm.

San Roman et al. discovered that the human Y chromosome and the inactive X chromosome exert modulatory effects on the expression of autosomal genes (79). Their findings that variations in the copy numbers of sex chromosome can influence autosomal gene expression. Further analysis revealed that ZFX and ZFY act in concert to modulate the expression of a part of autosomal genes (79).

Upon comparison with the specific proteins identified by Shen et al., it was observed that the genes encoding the specifically expressed protein SCAMP1 and the differentially expressed proteins Kidins220 and STX2 in X-/Y- sperm are targets of ZFX. In fibroblasts (XY) of the ZFY and ZFX knockouts, the expression levels of SCAMP1 were significantly upregulated 14% and 7.5%, respectively, whereas the expression of Kidins220 and STX2 showed no significant changes (26, 79). These results suggest that the specific expression patterns of unique proteins in X-/Y- sperm may be subject to regulation by ZFY or ZFX. Also, the regulatory mechanisms remain to be elucidated. It is important to note that San Roman study was conducted using somatic cells, and thus the regulatory and outcomes in germ cells may potentially differ (79). The results of this study also provide a new direction for research on X/Y sperm differences.

The identification of sex-specific molecular markers in X and Y sperm has been challenging owing to technical constraints in the past decades. The primary methods included the analysis of human disease cases and gene knockout within model organisms. These methods progressed indistinctly and identified only a limited number of genes and proteins with sex-specific expression in animal sperm. However, in recent years, multi-omics analysis has been applied in the study of sex-specific gene expression in sperm. Particularly, the introduction of proteomic analysis has facilitated the identification of sex-specifically expressed proteins. A number of differentially expressed proteins, which possess X or Y specifically expression, were identified through comparative proteomic analysis based on the sexed sperm (24–26). Identification of surface proteins specifically expressed in X and Y sperm offers a few potential targets for immunological sorting of bovine sperm (26). Moreover, the identification of sex-specifically expressed proteins provides potential targets for sperm sorting with immunomodulator and the sperm swim-up method (27, 28), or producing male- or female-only litters with 100% efficiency via gene-editing technology (80). Thus, the identification of unique proteins in X or Y sperm via proteomic analysis is the primary focus of sperm sorting with antibodies, small molecule inhibitors, and gene editing technologies.

In 1997, Howes et al. used SDS-PAGE and two-dimensional gel electrophoresis (2-DE) to analyze the protein profile of X and Y chromosome-bearing bull spermatozoa, with the aim of identifying differentially expressed proteins (DEPs) (81). Howes and colleagues provided firstly a route to identify DEPs in X and Y sperm using proteomics, paving the way for future investigations. Subsequently, 2DE and mass spectrometry (MS) were used to study the proteome of mammalian sperm. This approach led to the identification of several differentially- and specifically-expressed proteins in X and Y sperm (82). In the past two decades, promising results have been achieved in the identification of proteins in sperm. Sang et al. (83) from the Animal Genetics and Breeding team at Huazhong Agricultural University in China reported that they immunized rabbits with sexed bovine sperm to obtain antisera. Purified serum antibodies were then used to detect X- or Y-proteins in combination with 2DE to screen for sex-specific proteins of bovine sperm, and a protein of approximately 30 kD, specific to X sperm, was identified (83, 84). Chen et al. (85) from the Chinese Academy of Agricultural Sciences used 2DE to separate proteins of sexed bull X and Y sperm and identified 42 protein spots that showed differential expression between X and Y spermatozoa. Of these, 16 DEPs were detected using mass spectrometry. One of the proteins, 2EIN_R (R Chain R, zinc ion binding structure of bovine heart cytochrome c oxidase in the fully oxidized state), was found exclusively in X sperm (85). De Canio et al. from the University of Milan in Italy, identified two proteins upregulated in bovine Y sperm and an additional 15 proteins that were in X sperm through comparative proteomic analysis (86).

In recent years, there have been significant advancements in the identification of differentially expressed proteins in bovine sperm. Scott et al. from the University of São Paulo in Brazil, revealed eight proteins with differential expression between X and Y sperm in bulls using mass spectrometry (24). Additionally, Sharma et al. from G. B. Pant University of Agri. & Tech, India, identified 113 DEPs in X- and Y- bull semen using nano LC–MS. Among these, six proteins including NDC1 transmembrane nucleoporin, Beta-nerve growth factor, C-type natriuretic peptide, Nucleobindin 2, Phosphoglycerate mutase 2 and Calmodulin exhibited the potential as biomarkers for sperm sorting (25). Despite identifying multiple DEPs through proteomic analysis, the papers did not describe any further experimental validation of these proteins in X or Y sperm. Significantly, Shen et al. (26) from China Agricultural University investigated the differential expression of total membrane proteins in two types of sperm by using high-purity X- and Y-sperm from 20 Holstein bulls. They identified a total of 1,521 proteins with 8 proteins up-regulated and 23 proteins down-regulated in X sperm. Additionally, 151 and 88 proteins were specifically expressed in the X- and the Y- sperm, respectively. By Western blot analysis, the CLRN3 and SCAMP1 proteins were verified as cell surface specific antigens of X- and Y-sperm, respectively (26). These unique membrane proteins provide potential targets for immunological sorting of sperm. Moreover, CLRN3 and SCAMP1 proteins can be used as positive controls to assist identification of X and Y sperm-specific proteins. Notable achievements have been made in the analysis of the sperm proteome, hundreds of sperm cell membrane proteins were identified (87, 88), and Quelhas et al. (82) provided a comprehensive review of these achievements (82). In addition to cattle, X/Y sperm specific proteins in swine have also been reported. Cheng et al. from Shaanxi University of Technology identified COX6A1 and CYTB as possible antigens specific to swine Y sperm (89). Furthermore, transcriptomics and proteomics provide new platforms to investigate the composition and expression of X and Y sperm genomes. Three recent review articles offered comprehensive overviews of the latest relevant outcomes and advancements (3, 90, 91). Here, a compilation of the differentially or specifically expressed sperm proteins in X and Y sperm obtained from bovine is presented in Table 2.

**Table 2:** Differentially-expressed or sex-specifically-expressed proteins elucidated by proteomic analysis of bovine sperm.

Protein name	Gene name	Expression pattern (X. vs. Y.)	Reference
FUN14 domain-containing protein 2	FUNDC2	↑	Scott et al. (24)
Sorting and assembly machinery component 50 homolog	SAMM50	↑	
EF-hand domain-containing protein 1	EFHC1	↓	
Pyruvate dehydrogenase protein X component	PHDX	↓	
Outer dense fiber protein1	ODF1	↑	Sharma et al. (25)
Fructose-1,6-bisphosphatase 1	FBP1	↑	
Calmodulin	CALM	↑	
Glyceraldehyde-3-phosphate dehydrogenase, testis-specific	GAPDHS	↑	
Plasminogen	PLG	↓	
Zinc finger protein 292	ZNF292	↓	
Serotransferrin	TF	↓	
CLRN3 protein	CLRN3	↑^*^	Shen et al. (26)
Secretory carrier-associated membrane protein	SCAMP1	↓*	
Isocitrate dehydrogenase 3 (NAD^+^) α	IDH3A	↑	Chen et al. (85)
Cytochrome b-c1 complex subunit 1, mitochondrial	UQCRC1	↑	
Neutral sphingomyelinase activation-associated factor	NSMAF	↑	
Glutathione-S-transferase, μ3 (brain)	GSTM3	↓	
ATP synthase subunit β, mitochondrial	ATP5B	↓	
F-actin-capping protein subunit β	CAPZB	↓	
Glyceraldehyde 3 phosphate dehydrogenase	GAPDH	↑	De Canio et al. (86)
Outer dense fiber protein 2	ODF2	↑	
L-lactate dehydrogenase A	LDHA	↑	
Outer dense fiber protein 1	ODF1	↑	
A kinase anchor protein 3	AKAP3	↑	
Glyceraldehyde 3 phosphate dehydrogenase testis-specific	GAPDHS	↑	
Sperm acrosome membrane-associated protein 1	SPACA1	↑	
Triosephosphate isomerase	TPI1	↑	
Calmodulin	CALM	↑	
Tubulin alpha 8	TUBA8	↓	
Tubulin beta 2B	TUBB2B	↓	

* Unique expression in sperm.

## 5 Immunomodulator R848 for sperm sorting

R848 (Resiquimod, S28463) is an imidazoquinoline compound that acts as an immunomodulator that causes a severe inflammatory reaction (92). Importantly, R848 is a selective ligand for Toll-like receptor 7 (TLR7) and TLR8 with mixed TLR7/8 agonist activity (93). TLR7 and TLR8 are encoded on the X chromosome in mice, humans, sheep and cattle according to the GenBank database.^1^ Umehara et al. (27) from Hiroshima University in Japan, screened the sperm transcriptome RNA sequence database (DAR007935, https://www.ncbi.nlm.nih.gov/sra/?term=%20DRA007935) from 12-week-old male C57/BL6 mice and identified 18 X-chromosome-encoded gene products as receptors. Further analysis revealed that six of these receptors, namely TLR7, TLR8, Ar, GPR174, GPR34, and Edr2a, have specific ligands. Using RT-PCR, western blot, flow cytometry, and immunofluorescence, products of TLR7/8 were shown to be specifically expressed in mouse X sperm. TLR7 was expressed in the flagella of sperm, while TLR8 in the midpiece of sperm (27). This critical discovery provides new targets for sorting sperm.

R848, a specific dual agonist for TLR7/8, was utilized by Umehara and colleagues to treat mice or frozen–thawed bull sperm. The motility of X sperm declined in the sperm swim-up buffer as a result of R848 treatment (27, 28). TLR7/8 are known as endosomal membrane proteins and function as pattern recognition receptors (PRRs) that detect intracellular pathogen-associated molecular patterns (PAMPs) (94). The activation of TLR8 localized in the midpiece of sperm suppressed mitochondrial activity, while the activation of TLR7 localized in the tail of sperm suppressed cytoplasmic glycolysis, resulting in slow mobility of X sperm, which were subsequently found in the lower layer after a swim-up test with TLR7/8 ligand. Following *in vitro* fertilization using ligand-selected high-mobility mouse sperm, 90% of the embryos were XY males. Likewise, 83% of the pups obtained following embryo transfer were XY males. Conversely, the TLR7/8-activated sperm with slow mobility sperm produced embryos and pups, 81% of which were XX females (27). The application of a protein with an established role in innate immunity and its specific agonist for sperm sorting represents an innovative concept. This establishes a comprehensive system for sperm sorting and the pre-determination of the sex of early embryos through treatment with an immunomodulator. A simple sperm-sexing method was established for the efficient production of sexed mouse or cattle embryos through activating TLR7/8 on X sperm (27, 28).

By means of R848 and the sperm swim-up method, scholars from Northwest A&F University in China reported their achievements obtained from sperm sorting and sexed early embryos of cattle and dairy goats (95–100). Semen collected from Qinchuan cattle or Guanzhong dairy goat bucks was treated with R848 in sperm swim-up buffer. High-motility Y sperm segregated to the upper layer, while the low-motility X-sperm segregated to the lower layer. Flow cytometry was applied to count the rate of X and Y sperm, the percentage of upper Y sperm was 88.6%, and the lower X sperm was 72.5% in cattle (95). Following *in vitro* fertilization using TLR7/8-activated sperm from the lower layer, 80.52% of the embryos were XX females in dairy goats detected by RT-PCR (96). Additionally, they reported the effects of pH, Ca2^+^, and the combination of pH with R848 on sperm motility in dairy goats. This research was subsequently utilized for sperm sorting and sex pre-determination of offspring (97–100). When dairy goat semen was treated with R848 at pH 7.4 and separated using the sperm swim-up method, the percentage of lower X sperm reached 85.57%. Using the X sperm-rich seminal fluid for IVF resulted in a female embryo rate of 83.25%, with a subsequent 62.79% female offspring achieved via artificial insemination (97). Here, a compilation of R848 concentrations and pH values for sperm sorting in mouse, cattle and dairy goat is presented in Table 3.

**Table 3:** The concentration of R848 used to sort X-, Y-sperm in sperm swim-up buffer.

Species (spermatozoa)	R848		Efficiency		Reference
		Sperm	Embryos	Pups	
Mice	0.3 μM	˃90%Y	89.6%XY 69.8%XX	83.1% XY 81.4% XX	Umehara et al. (27)
Mice	0.03 μM^*^	86.2% X	˃90% XY ˃80% XX	/	Umehara et al. (28)
Bovine	0.03 μM^*^	/	˃90% XY ˃80% XX	/	Umehara et al. (28)
Bovine	0.6 μM	88.6% Y 79.3% X	/	/	Wen et al. (95)
Goat	1.0 μM	90.5% Y80.3% X	80.5% XX88.9% XX	/	Ren et al. (96)
Goat	0.2 μg/ml	85.6% X	83.3% XX	62.8% XX	Huang et al. (97)

* R848 dissolved in DMSO.

The utilization of R848 for the separation of X/Y sperm appears to be a promising approach; however, several potential issues associated with R848 warrant further discussion. Firstly, the addition of creatine, which was introduced by Umehara to enhance the separation efficacy of R848 in mouse and cattle X/Y sperm systems, has been observed to induce hyperactivation of sperm (101). The implications of pre-treating sperm with creatine for subsequent use in artificial insemination remain uncertain, particularly regarding potential effects on progeny. Notably, Ren and Wen successfully achieved high-purity separation of X/Y sperm in cattle and dairy goats without the supplementation of creatine, suggesting that creatine may not be an essential component for effective sperm separation (95, 96). Secondly, the stability of R848 is a matter of concern. While initial *in vitro* fertilization studies in goats reported a promising female offspring production rate of 83.3%, a subsequent decrease to 62.8% post-fertilization suggests a possible impact on sperm fertilization competence following R848 treatment (100). Despite the successful separation of X/Y sperm, the compromised motility of X sperm post-treatment may result in a diminished fertilization capacity, thereby skewing the sex ratio of the resulting offspring away from the expected proportion based on sex-selected sperm. Furthermore, the applicability of R848 is currently limited to a select few species. The separation of X/Y sperm using R848 has been demonstrated only in cattle and goats. In canines, despite the localization of the TLR7/8 gene on the X chromosome, no differential expression between X and Y sperm has been observed, and R848 has proven ineffective in separating X/Y sperm (102). Although canines are not typically considered dairy animals, this finding may hint at similar limitations in other dairy species, such as cows and sheep. Consequently, if R848’s utility is confined to a narrow range of animal species, traditional flow cytometry techniques may continue to represent the most efficacious method for X/Y sperm separation.

## 6 Comparison between different approaches of X/Y sperm separation

Following research spanning several decades, a multitude of techniques have been devised for the separation of X and Y spermatozoa. These methods for X/Y sperm sorting conclude albumin gradient centrifugation, Percoll density gradient centrifugation, immunomodulator R848 for sperm sorting, immunological separation, and flow cytometry. Each of these methodologies possesses distinct advantages, thereby offering a diverse array of tools for applications in sex selection and reproductive medicine.

Quinlivan WL et al. employed albumin gradients to effectuate the separation of X and Y spermatozoa. A fresh semen was introduced onto the upper layer of a 35% bovine serum albumin solution (103). Subsequent to a period of several hours, during which the spermatozoa were permitted to migrate, those spermatozoa that had settled at the base of the gradient were collected (103). Upon staining the collected spermatozoa with quinacrine hydrochloride, it was observed that the proportion of Y spermatozoa could be augmented to 74% (103). Brandriff et al. similarly utilized albumin gradients to isolate a population of spermatozoa that was 57.2% X spermatozoa (104). Furthermore, Aribarg et al. reported that albumin gradients exerted no discernible influence on the separation of X and Y spermatozoa (105). These findings underscore the inherent instability of albumin gradients. Additionally, it has been noted that albumin gradients are influenced by individual variability, with sperm separation efficiencies ranging from 6 to 37% (103). Moreover, a decline in sperm motility of 11% was observed following treatment with albumin gradients (103).

Kaneko et al. positioned spermatozoa on discontinuous Percoll density gradient, spanning a density range from 1.06 to 1.11 gm/mL. Post-centrifugation, at a density of 1.06 gm/mL, the concentration of Y spermatozoa was achieved to 73.1%, with a progressive diminution observed as the density increased (106). At the density of 1.11 gm/ml, the proportion of Y spermatozoa was reduced to 27.4% (106). Lin et al. constructed 12 distinct Percoll gradients for the purpose of separating X- and Y-spermatozoa. Fluorescence *in situ* hybridization (FISH) analysis revealed that Percoll was incapable of effectively separating X- and Y-spermatozoa (107). The conducted by Check indicated that spermatozoa subjected to Percoll density gradient centrifugation and albumin gradient centrifugation exhibited a reduced percentage of quinacrine-stained spermatozoa (108).

Upon a comparative analysis of these methodologies, the characteristics of each have been delineated. Both albumin gradient and Percoll gradient centrifugation are subject to instability, and the precision of sperm separation efficiency ascertained through quinacrine sperm staining remains equivocal. The issue of separation efficiency is also exist in R848 for sperm sorting. While the R848 is capable of sorting highly pure X- and Y-spermatozoa, its applicability is currently confined to a limited number of species, and it is with a decline in gender accuracy following artificial insemination. Flow cytometry represents the technology with the highest purity of X/Y sperm sorting and broadest market application; however, the prohibitive costs of equipment and patent fees limit its widespread adoption. The above methods methods each exhibit a unique set of advantages and disadvantages in the separation of X- and Y-spermatozoa. Immunological separation methods predicated on X- and Y-spermatozoa specific antigens have already yielded commercially available products, with separation efficiencies achieving 70%–80%. Consequently, the investigation of X- and Y-spermatozoa-specific proteins and the development of strategies to harness these proteins for immunological separation of X- and Y-spermatozoa warrant considerable attention.

## 7 Housekeeping proteins as Western blot loading control in sperm

Whether transcriptomic or proteomic is used to analyze gene products of sperm, the identification of specifically expressed proteins in X or Y sperm is the focus to the separation of X/Y sperm by immunological approaches. The identified specifically expressed proteins by proteomic analysis can be further validated with molecular and cellular biology techniques, including western blot, immunofluorescence, and immunohistochemistry (27, 95, 96). Western blotting is a commonly utilized immunodetection technique for the detection and (semi-) quantification specific proteins in provided samples. Significantly, western blotting is a relatively quantitative method. The expression level of the target protein is determined by comparing it with reference proteins (the internal loading control). In general, housekeeping proteins, which are encoded by housekeeping genes, are commonly utilized as loading controls to normalize target protein expression, including β-actin, glyceraldehyde 3-phosphate dehydrogenase (GAPDH), and tubulin. However, the expression of housekeeping proteins is not same in different kinds of sperm samples. Tubulin expression is consistent in both fresh and frozen semen in human sperm. In contrast, β-actin and GAPDH are relatively stable in fresh semen; nonetheless, their expression may fluctuate in frozen samples (109).

Tubulin is frequently used as a loading control in western blotting. However, different Tubulin isotypes exhibit varying expression patterns in X- and Y-sperm. In the context of mass spectrometry detection of bovine sperm tubulin, De Canio et al. (86) reported lower expression levels of tubulin-α3, tubulin-β4A, and tubulin-β4B in bovine Y sperm compared to X sperm. Conversely, the expression levels of tubulin-α8 and tubulin-β2B are higher in Y sperm than in X sperm (86), while tubulin-α1D and tubulin-α3D exhibit higher expression in X sperm than in Y sperm (25). In detection of sperm proteins using western blotting, tubulin was identified as a steady protein in sperm from mice (27), humans (109), bovine and goat (26, 95, 96, 100). The data obtained from both mass spectrometry and western blots indicates that tubulin, particularly β-tubulin, is a suitable reference protein.

Another frequently used loading control in western blotting experiments is the protein GAPDH. Chen et al. employed GAPDH as a reference protein in western blot analysis to validate the differential expression proteins in bovine X and Y sperm (85). Similarly, He et al. from Northwest A&F University, China, investigated target proteins in dairy goat sperm using GAPDH as an internal reference (99). However, mass spectrometry data by De Canio et al. reported that GAPDH up-regulated in bovine X sperm in comparison to Y sperm (86). Additionally, it has been documented that GAPDH was not detected in bovine Y sperm (25). Additionally, Kapitonova et al. from Moscow State University in Russia, revealed that GAPDH is consistently identified in bovine sperm, both in frozen–thawed conventional semen and frozen–thawed X sperm (110). Consequently, the findings from mass spectrometry and western blot analysis suggest that GAPDH may not be as suitable as tubulin for serving as an internal reference in comparative studies of protein expression in bovine X and Y sperm. It seems to be a more suitable choice for comparing protein expression between conventional sperm and sexed X sperm.

β-actin, an internal reference protein for western blot in somatic cells, is less frequently used in the assessment of sperm proteins. It was only detected in mouse sperm by Umehara et al. (27) and in buffalo sperm by Xiong et al. (111). Naresh (112) assessed the detection of total actin in fresh, refrigerated (4°C), and frozen bovine semen using western blot analysis. The study findings indicated that actin was present in the flagella and acrosome membrane regions of sperm before capacitation. Furthermore, the processes of cooling and cryopreservation were found to reduce the overall expression of actin (112). Moreover, the abundance of β-actin in fresh human semen remains relatively consistent, but it fluctuates significantly in frozen semen (109). For the comparison of protein expression among different treatments of sperm samples, the detection of reference proteins tends to be more efficient in fresh semen compared to frozen semen. β-tubulin (tubulin) is found to be a more suitable primary choice for a reference protein, with GAPDH as a secondary option. Finally, a loading control was suitably used to detect protein expression level of sperm samples, which can minimize loading variability, thereby revealing actual expressional differences. The characteristics of several reference proteins for western blot analysis of sperm protein were listed in Table 4.

**Table 4:** Internal reference proteins for western blot analysis of sperm proteins under different treatments.

	Fresh semen	Frozen semen	Sex sorted sperm
Tubulin	Yes (26)	Yes (27)	Yes (26)
β-actin	Yes (112)	No (112)	Instability
GAPDH	Yes (99)	No	Instability

## 8 Concluding remarks and future directions of immunological sorting of sperm

The generation of single-sex litters is a crucial factor in livestock production and is associated with economic benefits of dairy and meat processing. Sexing technology has been developed for the purpose of producing dairy calves on farms. Currently, flow cytometric sorting of X and Y sperm is the most widely applied method to generate the sex pre-determination of offspring. However, this technique still remains a technical bottleneck. The immunological sorting of spermatozoa has demonstrated high efficiency and practicality for pre-determination the sex of offspring in livestock.

Although the existence of cytoplasmic bridges during spermatogenesis enables X and Y sperm to share gene products, the fact that a large class of mammalian genes are not completely shared across these bridges makes it possible to find unique proteins in X or Y sperm. The immunological sorting approaches of sperm were discussed earlier. Whether sorting sperm with antibodies or immunomodulators is based on the identification of sex-specifically expressed proteins. Antibodies were used to sort sperm relies on membrane proteins with sex-specific expression, with SRY being the only reported antigen. However, it remains to be further determined whether SRY has characteristics of sperm surface expression and can be effectively applied to sperm sorting. The membrane proteins CLRN3 and SCAMP1 were potential targets for bovine X- and Y-sperm, respectively. At present, R848 is used for sperm sorting as an immunomodulator, with its target proteins being intracellular TLR7/8.

Herein, we have discussed several potential future directions for immunological methods in the separation of X/Y sperm. In 2011, Mohammadi et al. engineered polyclonal antibodies directed against the DBY peptide of swine, also recognized as DDX3Y. Utilizing these antibodies, they achieved the collection of over 90% of X spermatozoa (113). Comparative analyses via database alignment reveal a highly similar between sequence of DDX3X and DDX3Y. Therefore, how to enhance antibody specificity becomes a topic worthy of investigation. Antibodies or antiserum developed using Full length proteins as immunogens and through animal immunization might recognition proteins similar to antigens. Typically, the antigenic epitopes recognized by antibodies include 10–20 amino acids. The design of specific peptide, coupled with sequence alignment via database can enhance antibody specificity.

Hamers-Casterman identified a unique class of antibodies in camelids, lacked light chains and termed heavy-chain antibodies (HCAb) (114). These antibodies are characterized by a highly soluble variable immunoglobulin domain (VHH), linked via a hinge region to two constant immunoglobulin domains (CH2 and CH3) (115). This antibody is also known as a nanobody. Nanobodies represent a departure from monoclonal antibodies, with a molecular weight of approximately 15 kDa, facilitating their penetration through cell membranes and even traversal of the blood–brain barrier to exert therapeutic effects (114). A multitude of immunotherapeutic strategies leveraging nanobodies are currently in the developmental phase (103). The conventional approach of employing antiserum to sorting X/Y sperm is limited by the membrane-bound antigens, this is the reason why Shen et al. studied X/Y sperm membrane protein proteomics (26). Given the capacity of nanobodies to binding intracellular antigens, the scope of sperm membrane proteomics can be broadened to encompass the total sperm proteome, thereby expanding the avenues and methodologies for the separation of X-/Y- sperm. For instance, differential proteins associated with motility or energy metabolism, as identified through proteomic analysis of X-/Y- sperm, could be targeted with nanobodies to impair sperm function and facilitate separation.

Thus, it is anticipated that novel X or Y sperm-specific proteins will be discovered with the application of spermatozoa proteomics. The strategy employed to identify sex-specifically expressed proteins, followed by the implementation of immunological techniques to achieve sperm sorting, is currently being used to generate all-female or all-male offspring in livestock.

## Footnotes

^1^ www.ncbi.nlm.nih.gov/genbank
